# Design and Study of a Novel P-Type Junctionless FET for High Performance of CMOS Inverter

**DOI:** 10.3390/mi16010106

**Published:** 2025-01-17

**Authors:** Bin Wang, Ziyuan Tang, Yuxiang Song, Lu Liu, Weitao Yang, Longsheng Wu

**Affiliations:** State Key Discipline Laboratory of Wide Bandgap Semiconductor Technology, School of Microelectronics, Xidian University, Xi’an 710071, China

**Keywords:** PD-SOI, junctionless FET, buried layer, CMOS inverter

## Abstract

In this paper, a novel p-type junctionless field effect transistor (PJLFET) based on a partially depleted silicon-on-insulator (PD-SOI) is proposed and investigated. The novel PJLFET integrates a buried N+-doped layer under the channel to enable the device to be turned off, leading to a special work mechanism and optimized performance. Simulation results show that the proposed PJLFET demonstrates an I_on_/I_off_ ratio of more than seven orders of magnitude, with I_on_ reaching up to 2.56 × 10^−4^ A/μm, I_off_ as low as 3.99 × 10^−12^ A/μm, and a threshold voltage reduced to −0.43 V, exhibiting excellent electrical characteristics. Furthermore, a new CMOS inverter comprising a proposed PJLFET and a conventional NMOSFET is designed. With the identical geometric dimensions and gate electrode, the pull-up and pull-down driving capabilities of the proposed CMOS are equivalent, showing the potential for application in high-performance chips in the future.

## 1. Introduction

The scaling of metal oxide field-effect transistors (MOSFETs) to the nanometer regime faces several challenges, and the performance of PMOS becomes one of the key factors limiting the development of CMOS technology [1,2,3]. Since the mobility of holes is approximately half that of electrons, PMOS requires a larger size to compensate for its lower mobility to achieve the same current driving capability with NMOS, which affects the miniaturization of CMOS devices. To improve the mobility of PMOS devices and enhance the performance of CMOS devices, some studies focus on improving PMOS-driven capability by using lower bandgap materials [4,5,6,7]. However, the off-state current could rise simultaneously, leading to high standby power consumption. Moreover, the devices with hetero-materials have also posed a severe challenge to the fabrication process. Other studies try to enhance driving current with silicon-based PMOS by modifying structures [8,9,10,11]. Among them, the junctionless FET (JLFET) is one of the effective ways to improve the performance of PMOS devices.

**Figure 1:**
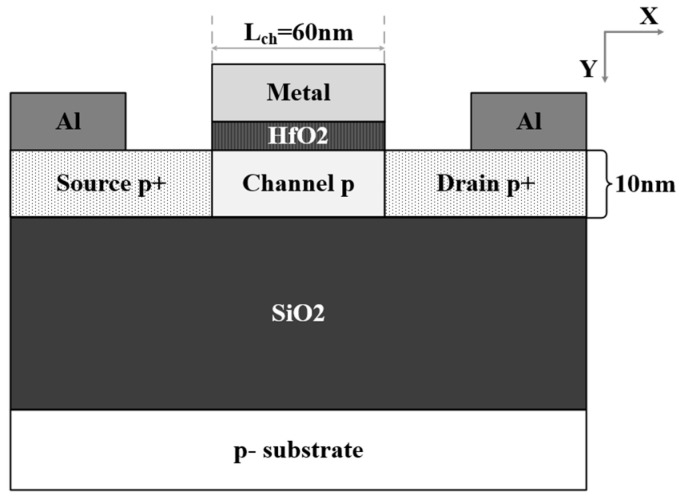
Cross-section of conventional PJLFET based on FD-SOI substrate.

Junctionless field effect transistors (JLFETs) have the advantages of higher carrier mobility, lower resistance and stronger driving ability [12,13,14,15] due to their special working mechanism. These JLFETs usually have the same doping type in the source, channel, and drain regions, so there is no PN junction along the current flow direction, as shown in Figure 1. Meanwhile, a large difference in work function between the gate electrode and channel is required to achieve volume depletion in the channel, shutting the device down in the off state. With an increasing gate bias voltage, the depletion region under the gate dies away from the bottom of channel to the surface of channel, and the entire channel region becomes neutral finally, allowing carriers to flow from source to drain. Due to the special work mechanism, the movement of charge carriers in the channel is subject to less scattering, enhancing their mobility significantly. Thus, JLFETs are characterized as bulk conductive devices and work in flatband mode or accumulation mode, and the driving ability is much better than that of conventional MOSFETs [16,17].

However, since the semiconductor film is heavily doped in JLFETs, to achieve volume depletion, the gate electrode work function of JLFET has to be adjusted to shut down the device in the off state. For p-type JLFET (p-JLFET), a low work function (≤4.1 eV) is required to achieve volume depletion under the gate in the channel region to completely inhibit majority-carrier current flow from source to drain. The gate electrode with low work function deteriorates the performance of the device: the surface electric field of the device in the off state is high, resulting in a serious gate-induced drain leakage (GIDL) effect which is the main component of the off-state current in JLFET [18,19,20], and a large gate voltage is required for the channel to exit the depletion state, causing a relatively high threshold voltage V_TH_ of p-JLFET. In addition, to realize efficient volume depletion of the channel in p-JLFETs, the silicon film is ultrathin (thickness ≤ 10 nm). The use of ultrathin silicon films, such as fully depleted SOI (FD-SOI), is costly [21]. Moreover, producing ultrathin silicon films with a uniform thickness over the entire wafer is a challenge [22].

Thus, in this paper, a novel p-type JLFET based on partially depleted SOI (PD-SOI) is proposed and studied for the first time. The novel PJLFET integrates a buried N+ doping layer under the channel to turn off the device, reducing the requirements for the gate electrode and the ultrathin silicon film. With the new structure, the proposed PJLFET is supposed to achieve both high on-state current (I_on_) and low off-state current (I_off_), keeping **V_TH_** relatively low at the same time. The performance of the proposed PJLFET is verified and studied by using a TCAD simulator. Simulation results show that the proposed PJLFET demonstrates an I_on_/I_off_ ratio of more than 7 decades of magnitude with I_on_ up to 2.56 × 10^−4^ A/μm, I_off_ down to 3.99 × 10^−12^ A/μm, and V_TH_ is −0.43 V. Furthermore, a new CMOS inverter is designed. The new CMOS is composed of a proposed PJLFET and a conventional NMOSFET, and the voltage transfer characteristic (VTC) and output transient characteristic are examined in detail.

## 2. Device Structure and Simulation Approach

**Figure 2:**
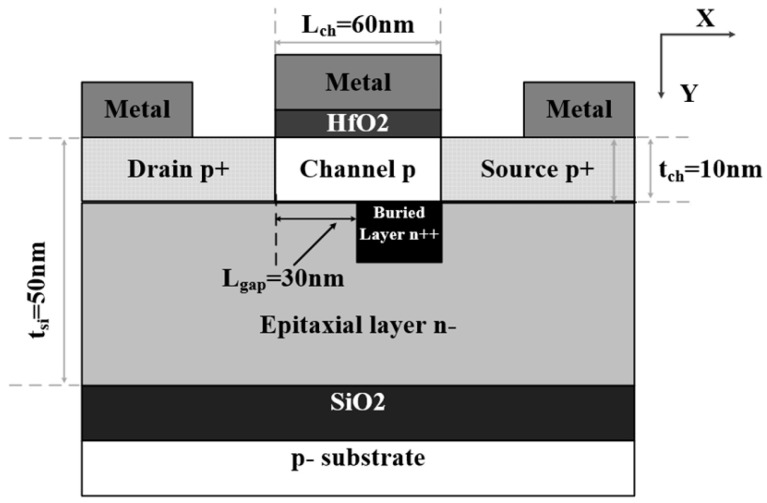
Cross-section of BNL PJLFET based on PD-SOI substrate.

Figure 2 shows the cross-section of the proposed p-type JLFET with a buried N+-doped layer (BNL PJLFET) based on PD-SOI. Different from conventional PJLFETs, the proposed BNL PJLFET has a buried N+ doped layer (BN layer) under the channel, and an electrode with a work function of 4.8 eV is used for the JLFET gate metal. Due to the heavily doped BN layer, a higher point-tunneling probability occurs between the BN layer and drain in the off state. So, the gap (L_gap_) between the BN layer and the edge of the gate electrode is introduced to restrict the point tunneling at zero gate bias. In addition, the channel is lightly doped compared with the BN layer to ensure its depletion, and the high k material of HfO_2_ is used as gate oxide dielectric. The parameters used in our simulation are shown in Table 1.

The simulation is carried out using the Sentaurus TCAD simulator. The simulation models used in [23] were implemented in our simulations. The dynamic nonlocal band-to-band tunneling (BTBT) model is used for BTBT current simulation, while the bandgap narrowing effect and Fermi statistical distribution are also taken into account. The involved parameters are carefully calibrated according to [24,25]. Since the effective bandgap directly influences the tunneling current, the bandgap narrowing (BGN) model is also included. The Shockley–Read–Hall (SRH) recombination model is included due to the presence of high-impurity atom concentration in the channel and the Fermi–Dirac statistics are used to calculate the intrinsic carrier concentration. For more accurate current calculations, the field-dependent, doping-dependent mobility degradation model, and drift-diffusion current transport model, are also considered.

## 3. Operation Principle of BNL PJLFET

**Figure 3:**
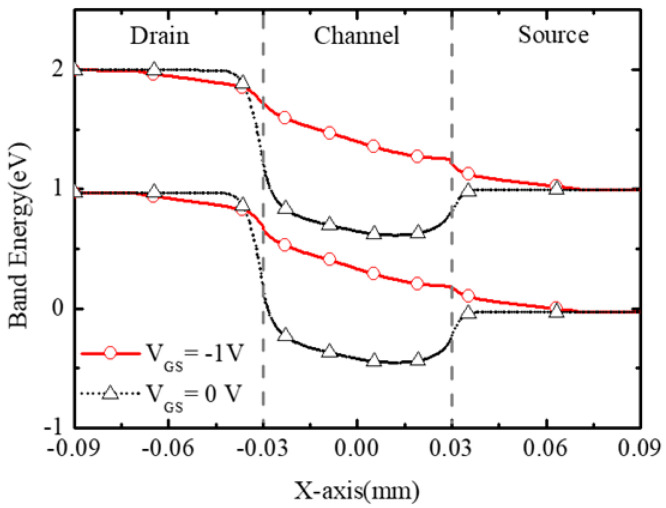
Band diagrams of BNL PJLFET along the surface of the channel.

The device operation can be understood from the band diagrams in BNL JLFET illustrated in Figure 3. The simulated band diagrams are along the surface of the channel as shown in Figure 2.

At zero gate bias (V_GS_ = 0 V), since the depletion region of the n+/p− junction is mainly distributed on the lightly doped p region, the channel above the BN layer is depleted. So, there is a high barrier (for holes) between the drain and channel, as shown in Figure 3. Thus, the device works in the off state and the current cannot flow from the source to drain.

As the gate bias voltage │V_GS_│ increases, the hole barrier gradually decreases. Finally, the surface of the channel exits the depletion state and enters a neutral state finally, causing the device to turn on (as shown in Figure 3). Since the depletion state caused by the buried layer is weaker closer to the surface, the holes appear from the surface to the bottom of the channel gradually, being different from the operation of the conventional PJLFETs. At V_GS_ = −1 V, a bulk hole channel is formed, and the holes can flow from source to drain at V_DS_ = −1 V.

## 4. Results and Discussion

**Figure 4:**
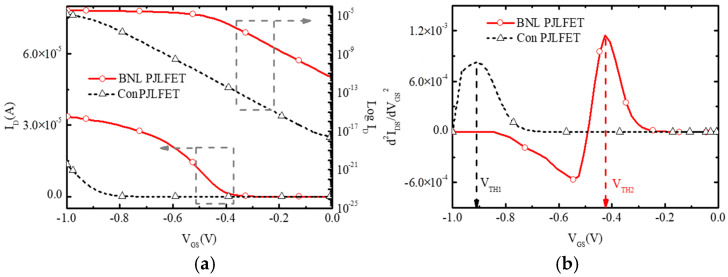
Extraction of V_TH_ of BNL PJLFET and conventional PJLFET. (**a**) Transfer characteristics at V_DS_ = −0.05 V (**b**) d^2^I_DS_/dV_GS_^2^ curves.

To extract the threshold voltage V_TH_, the transfer characteristics of the proposed BNL PJLFET and conventional PJLFET at V_DS_ = −0.05 V are obtained, as shown in Figure 4a. To deplete carriers in the channel with a thickness of 10 nm, the conventional PJLFET has a gate electrode with a 4.1 eV work function to shut down the device. A transconductance derivative method is used to extract V_TH_ of JLFETs from transfer curves. Figure 4b calculates the d^2^I_DS_/dV_GS_^2^ curves as a function of V_GS_. It can be seen that the maximum d^2^I_DS_/dV_GS_^2^ values of the proposed BNL PJLFET and conventional PJLFET are obtained at V_GS_ = −0.43 V and V_GS_ = −0.91 V, which indicates V_TH_ of BNL PJLFET is much smaller than that of conventional PJLFET, as a result of the increase in work function of the gate electrode as mentioned above.

**Figure 5:**
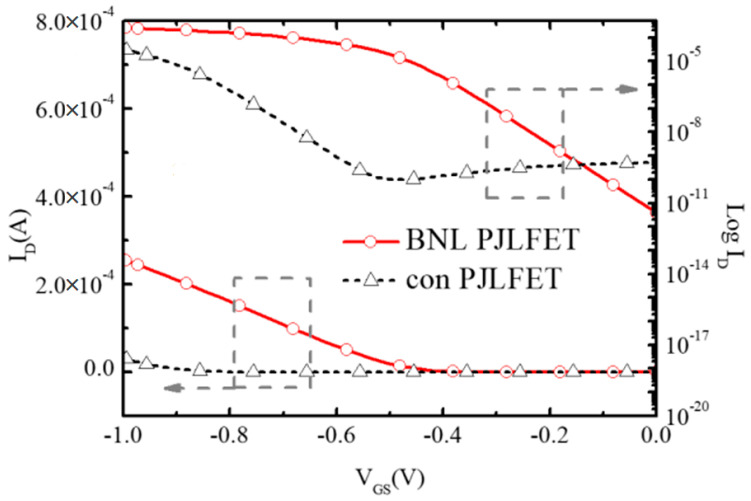
Transfer characteristic of the BNL PJLFET and the conventional PJLFET at V_DS_ = −1 V.

Figure 5 shows the transfer characteristics of conventional PJLFET and the proposed BNL PJLFET at V_DS_ = −1 V. As discussed above, the V_TH_ of BNL PJLFET is lower than that of conventional JLFET. Thus, the on-state current I_on_ of BNL PJLFET extracted at V_GS_ = −1 V obtains a value of 2.56 × 10^−4^ A/μm, which is larger than the value of 3.17 × 10^−5^ A/μm extracted in conventional PJLFET. Meanwhile, off-state current I_off_ of BNL PJLFET at V_GS_ = 0 V is 3.99 × 10^−12^ A/μm, which is two orders of magnitude lower than that in conventional PJLFET, leading to a high I_on_/I_off_ ratio of 6.42 × 10^7^.

**Figure 6:**
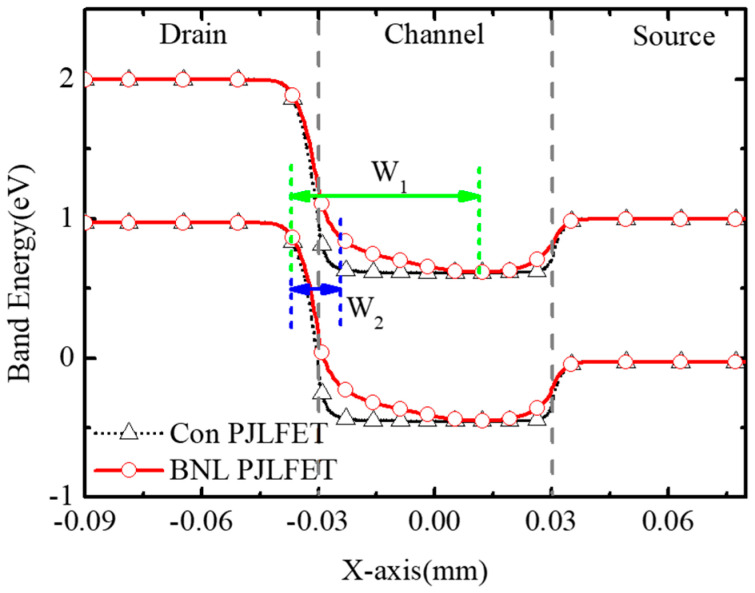
Tunneling width at the channel surface of the BNL PJLFET and the conventional PJLFET at V_GS_ = 0 V, V_DS_ = −1 V.

Figure 6 shows the evidence of the GIDL suppression in the off state (V_DS_ = −1 V, V_GS_ = 0 V) of the BNL PJLFET. W_1_ and W_2_ represent the tunneling width in BNL JLFET and conventional JLFET, respectively. Due to the optimization of the work function of the gate electrode and the introduction of the gap between the BNL and the drain, the tunneling width W_1_ at the drain–channel interface of BNL JLFET is significantly larger than that of conventional JLFETs. An enlarged tunneling width leads to a reduction in the tunneling probability of electrons from the drain region to the channel region, as pointed out in the literature [26], and decreases the off-state current I_off_ in the BNL PJLFET.

**Figure 7:**
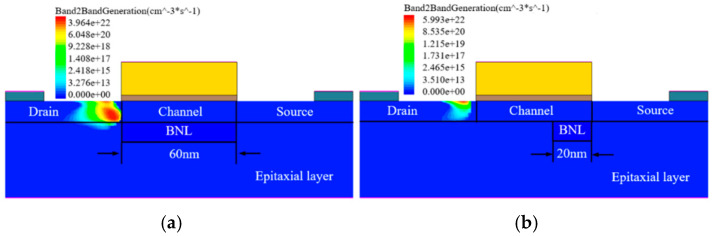
Band to band tunneling probability distribution at the surface of BNL PJLFET with (**a**) L_BN_ = 20 nm and (**b**) L_BN_ = 60 nm when V_GS_ = 0 V, V_DS_ = −1 V.

As discussed in Section 2, there is a significant band to band tunneling between the drain region and the BN layer. Figure 7 shows the BTBT probability with L_BN_ = 60 nm and 20 nm, respectively. It can be seen that a significant BTBT occurs at the drain–BN junction at L_BN_ = 60 nm. To suppress the BTBT effect between the drain and BN layer, the gap between the drain and BNL is introduced. With the decrease of L_BN_, the highest BTBT probability point moves from the interior of the device to the surface at the interface of the drain and channel, eliminating the influence of bulk tunneling on the off-state current.

**Figure 8:**
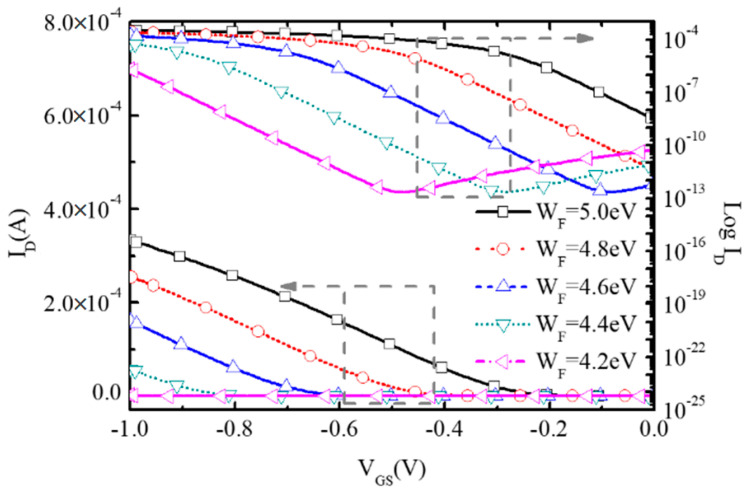
Transfer characteristic of the proposed BNL PJLFET for different work function at V_DS_ = −1 V.

Figure 8 shows the influence of the work function (WF) of the gate electrode on the transfer characteristics of BNL JLFET. With the reduction in WF from 5.0 eV to 4.2 eV, the GIDL effect becomes more and more significant, resulting in the increase of I_off_ from 8.51 × 10^−12^ A/μm to 3.04 × 10^−9^ A/μm.

**Figure 9:**
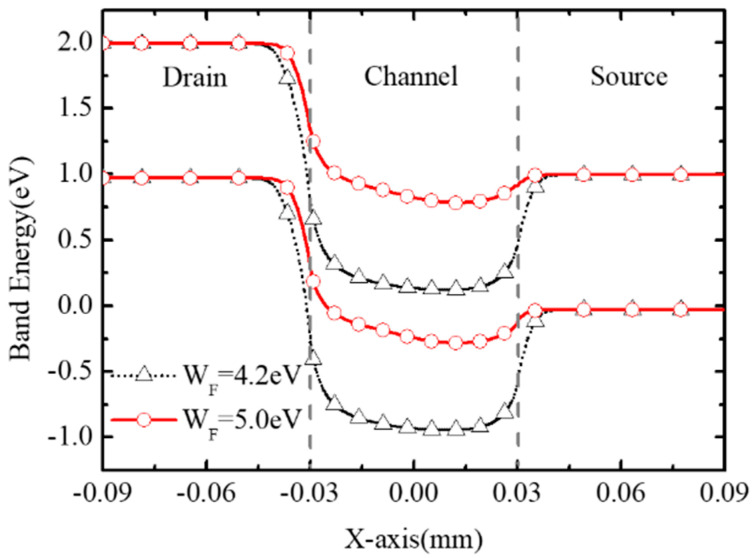
Band diagrams along the surface of the channel with WF = 4.2 eV and 5.0 eV when V_GS_ = 0 V, V_DS_ = −1 V.

Figure 9 shows the band diagrams of BNL PJLFET for WF = 5.0 eV and 4.2 eV, respectively. It can be seen that the BTBT width at the drain–channel interface with WF = 4.2 eV is narrower than that of the device with WF = 5.0 eV, resulting in the enhancement of band to band tunneling from the drain to channel. Thus, the GIDL effect is enhanced and I_off_ increases. Moreover, since the depletion degree in the channel improves as WF changes from 5.0 eV to 4.2 eV, the threshold voltage also increases, resulting in the reduction in on-state current I_on_.

**Figure 10:**
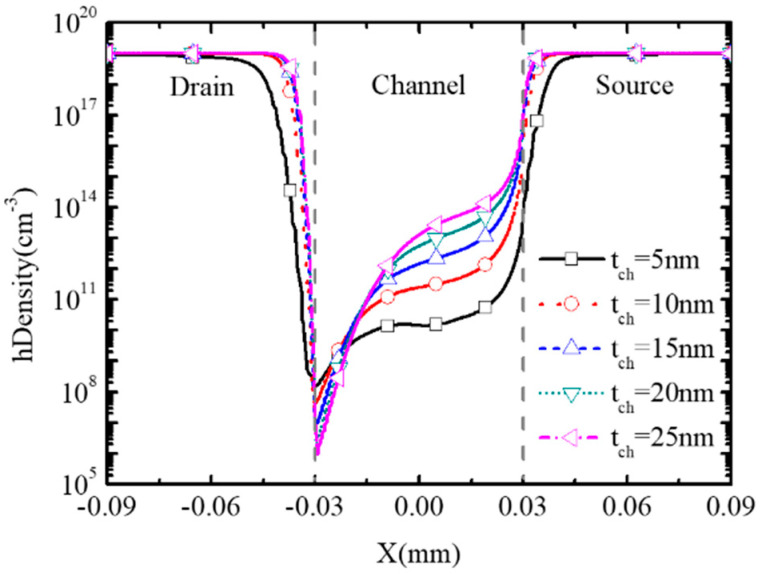
Surface hole density of BNL pJLFET for different tch at V_GS_ = 0 V, V_DS_ = −1 V.

**Figure 11:**
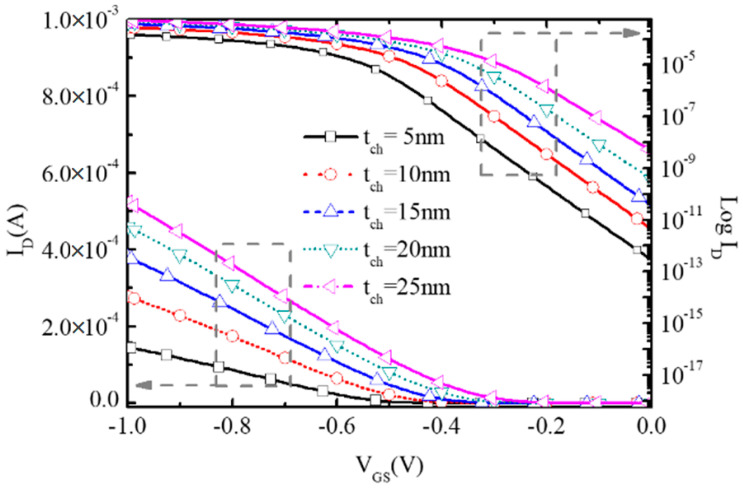
Transfer characteristic of the proposed BNL PJLFET for different t_ch_ at V_DS_ = −1 V.

To study the impact of channel thickness t_ch_ on the performance of BNL PJLFET, Figure 10 examines the surface hole density with tch changing from 5 nm to 25 nm at V_GS_ = 0 V, V_DS_ = −1 V. Since the channel is depleted by the buried n^+^/p^−^ junction, a large t_ch_ means the reduction in depletion degree at the channel surface. So, at an off state, the surface hole density at t_ch_ = 25 nm is higher compared with that at t_ch_ = 5 nm, resulting in the increment of off-state current I_off_ from 2.85 × 10^−13^ A/μm to 4.89 × 10^−9^ A/μm, as shown in Figure 11.

**Figure 12:**
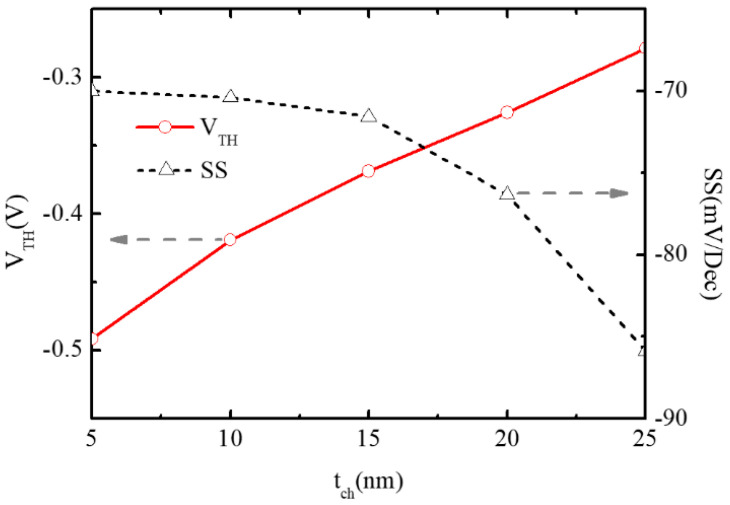
Variations of V_TH_ and SS of BNL PJLFET with t_ch_ changing from 5 nm to 25 nm.

Owing to the reduction in surface depletion degree, the absolute value of threshold voltage V_TH_ decreases with the increase in the channel thickness as shown in Figure 12. So, the on-state current I_on_ at V_GS_ = −1 V, V_DS_ = −1 V increases to 5.23 × 10^−4^ A/μm at t_ch_ = 25 nm, almost three times larger than 1.47 × 10^−4^ A/μm at t_ch_ = 5 nm shown in Figure 11. However, as the influence of the increment of the channel thickness on off-state current I_off_ is more apparent, it can be seen that the subthreshold swing SS deteriorates with the enlargement of the channel thickness in Figure 12.

**Figure 13:**
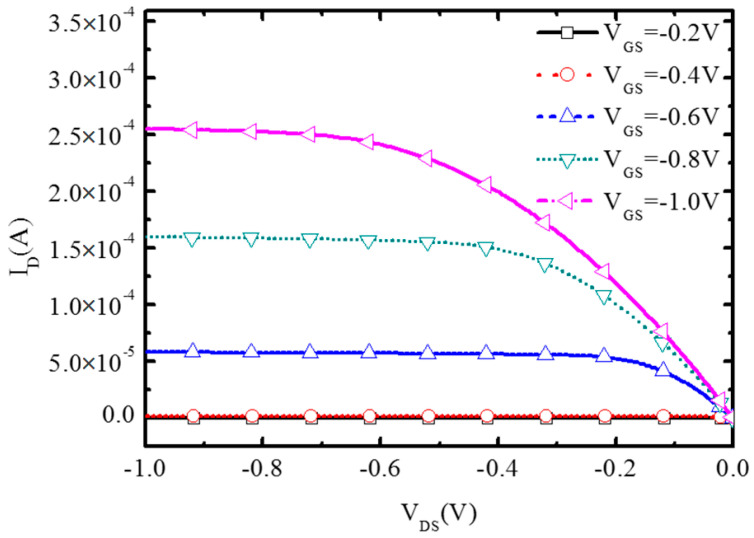
BNL PJLFET output characteristic curves.

Figure 13 shows the output characteristics of the BNL PJLFET with different gate voltages. The drain current I_DS_ at V_GS_ = −0.2 V, −0.4 V, −0.6 V, −0.8 V, and −1 V increases firstly and then reaches saturation at high drain voltages, showing good conduction characteristics.

## 5. Investigation

**Figure 14:**
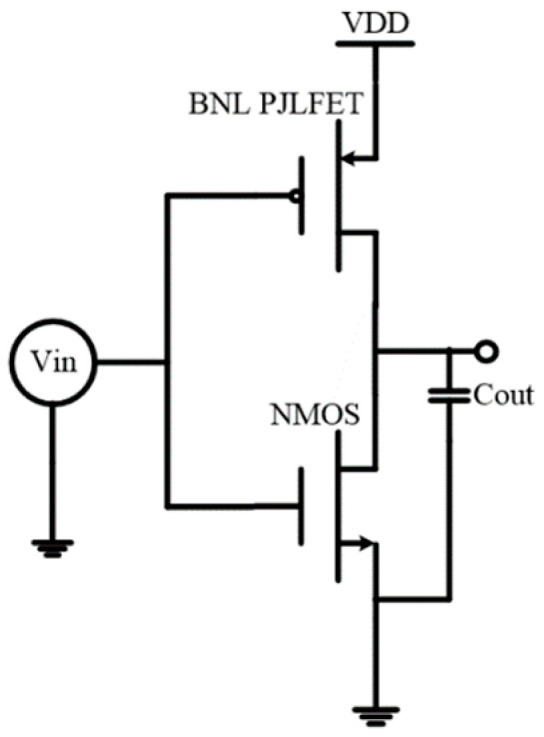
Inverter circuit in Sentaurus TCAD.

The investigation of the BNL PJLFET in the CMOS inverter is examined in this section. As shown in Figure 14, the new CMOS inverter consists of a BNL PJLFET as the pull-up device and a conventional NMOS as the pull-down device, where the BNL PJLFET operates in the accumulation region and the conventional NMOS operates in the inversion region. This is completely different from the conventional CMOS devices based on the inversion model. C_out_ is the load capacitance with the value of 1 × 10^−14^ F. The parameters of proposed CMOS used in the simulation are shown in Table 2. Moreover, the geometric dimensions of PJLFET and NMOSFET are identical in the new CMOS inverter, and the same metal electrode is used for both devices.

**Figure 15:**
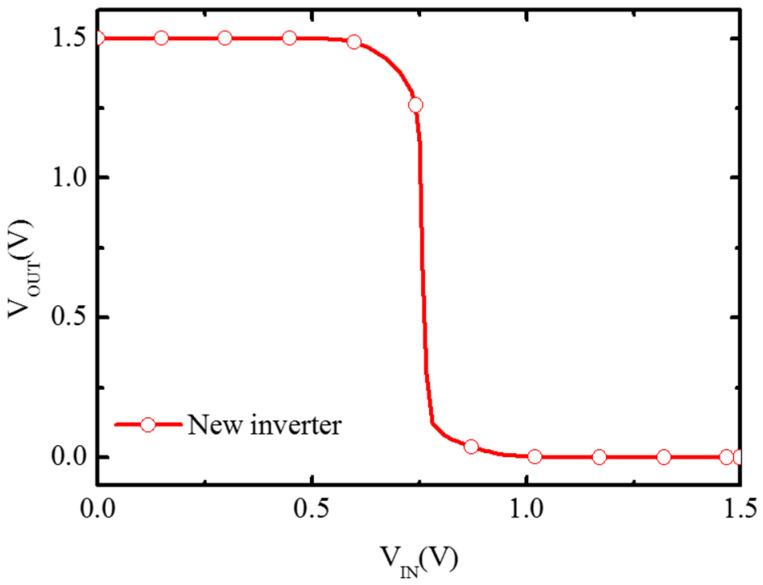
VTC curve of new inverter.

**Figure 16:**
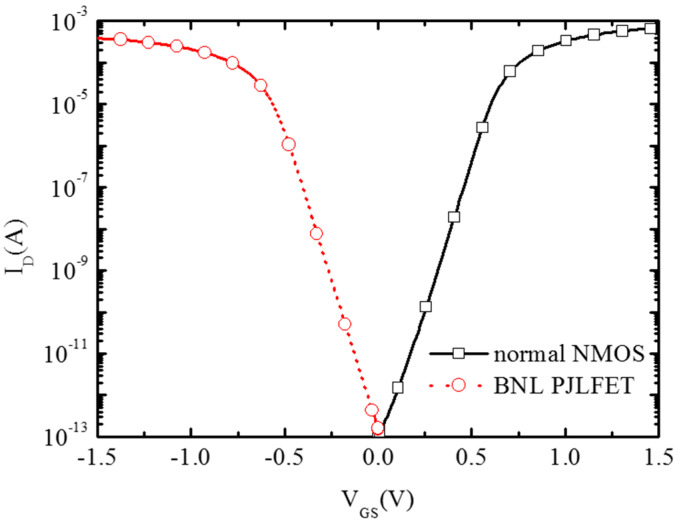
Transfer characteristic curves of the devices in the inverter.

Figure 15 shows the VTC (Voltage Transfer Characteristic) curve of the inverter through simulation. It can be seen that the threshold conversion voltage VM of this new inverter is almost at the middle voltage of V_IN_, showing a good transfer characteristic of the CMOS inverter. This is mainly attributed to the symmetrical performance of conventional NMOS and BNL PJLFET as shown in Figure 16. Figure 16 shows the transfer characteristic curves of BNL PJLFET and conventional NMOS at V_DS_ = 1 V, respectively. The curves of conventional NMOS and BNL PJLFET are almost symmetrical. The extracted threshold voltages of these two devices are -0.45 V and 0.47 V, respectively, showing the evidence for the symmetrical performance of PJLFET and conventional NMOSFET.

## 6. Conclusions

A novel p-type JLFET based on PD-SOI with a buried N+-doped layer under the channel (BNL PJLFET) is proposed in this paper. By introducing a heavily doped N+ buried layer, the device is shut down by the buried p/n junction. Using Sentaurus TCAD tools, the simulation results indicate that BNL PJLFET could effectively suppress the GIDL effect and reduce the threshold voltage, resulting in the improvement of on-state current and on/off current ratio. Furthermore, a new type of CMOS inverter with the BNL PJLFET as a pull-up device was proposed and examined; simulation results show the evidence of performance enhancement of the new CMOS inverter.

## Figures and Tables

**Table 1:** Simulated device parameters used in this study.

Parameter	BNL PJLFET	CON PJLFET
Gate length (L_ch_)	60 nm	60 nm
Channel doping concentration	1 × 10^18^ cm^−3^	1 × 10^18^ cm^−3^
HfO_2_ thickness (T_ox_)	3 nm	3 nm
Buried layer length (L_BN_)	30 nm	/
Epitaxial layer doping (N_epi_)	1 × 10^16^ cm^−3^	/
Buried performing concentration (N_BN_)	1 × 10^19^ cm^−3^	/
Work function of gate electrode	4.8 eV	4.1 eV
Work function of source/drain electrode	4.1 eV	4.1 eV
Source/drain doping concentration	1 × 10^20^ cm^−3^	1 × 10^20^ cm^−3^

**Table 2:** Device parameters used in these CMOS inverters.

Parameter	BNL PJLFET	CON NMOS
Gate length (L_ch_)	60 nm	60 nm
Channel doping concentration	1 × 10^18^ cm^−3^	1 × 10^18^ cm^−3^
HfO_2_ thickness (T_ox_)	3 nm	3 nm
Buried layer length (L_BN_)	30 nm	/
Epitaxial layer doping (N_epi_)	1 × 10^16^ cm^−3^	1 × 10^16^ cm^−3^
Buried performing concentration (N_BN_)	1 × 10^19^ cm^−3^	/
Work function of gate electrode	4.7 eV	4.7 eV
Work function of source/drain electrode	4.1 eV	4.1 eV
Source/drain doping concentration	1 × 10^19^ cm^−3^	1 × 10^19^ cm^−3^

## Data Availability

The original contributions presented in the study are included in the article, further inquiries can be directed to the corresponding author.
